# mRNABench: A curated benchmark for mature mRNA property and function prediction

**DOI:** 10.1101/2025.07.05.662870

**Published:** 2025-07-08

**Authors:** Ruian (Ian) Shi, Taykhoom Dalal, Philip Fradkin, Divya Koyyalagunta, Simran Chhabria, Andrew Jung, Cyrus Tam, Defne Ceyhan, Jessica Lin, Kaitlin U. Laverty, Ilyes Baali, Bo Wang, Quaid Morris

**Affiliations:** 1 Department of Computer Science, University of Toronto; 2 Vector Institute; 3 Computational and Systems Biology Program, Sloan Kettering Institute; 4 Department of Electrical and Computer Engineering, University of Toronto; 5 Peter Munk Cardiac Center, University Health Network

## Abstract

Messenger RNA (mRNA) is central in gene expression, and its half-life, localization, and translation efficiency drive phenotypic diversity in eukaryotic cells. While supervised learning has widely been used to study the mRNA regulatory code, self-supervised foundation models support a wider range of transfer learning tasks. However, the dearth and homogeneity of standardized benchmarks limit efforts to pinpoint the strengths of various models. Here, we present mRNABench, a comprehensive benchmarking suite for mature mRNA biology that evaluates the representational quality of mature mRNA embeddings from self-supervised nucleotide foundation models. We curate ten datasets and 59 prediction tasks that broadly capture salient properties of mature mRNA, and assess the performance of 18 families of nucleotide foundation models for a total of 135K experiments. Using these experiments, we study parameter scaling, compositional generalization from learned biological features, and correlations between sequence compressibility and performance. We identify synergies between two self-supervised learning objectives, and pre-train a new Mamba-based model that achieves state-of-the-art performance using 700x fewer parameters. mRNABench can be found at: https://github.com/morrislab/mRNABench.

## 1 Introduction

Nucleotide foundation models show promise as general-purpose embedding models for RNA transcripts, offering rich representations useful for diverse RNA function prediction tasks. Despite an abundance of foundation models, and corresponding benchmarks for DNA [1, 2, 3, 4] and non-coding RNA (ncRNA) [5, 6], the modelling of messenger RNA remains underexplored.

**Figure 1:**
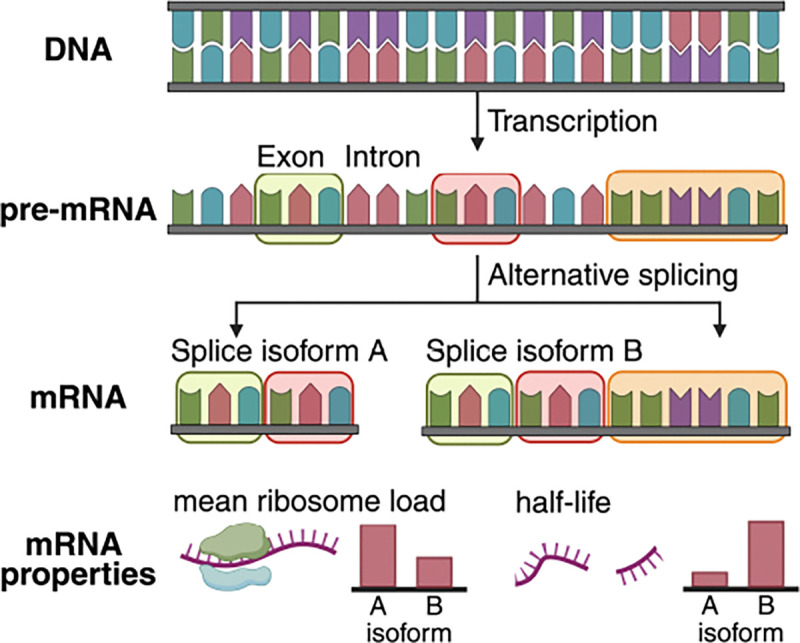
Genes are transcribed from DNA into pre-mRNA, which, through alternative splicing, can generate distinct mRNA isoforms, each with unique functional properties.

Mature messenger RNA (mRNA) is created by splicing which selectively retains exonic regions from a pre-mRNA creating an mRNA splice isoform. Alternative splicing is a combinatorial process that, from a single genomic locus, can generate multiple splice isoforms depending on exon choice, each with distinct properties and functions (Figure 1). Up to 90% of human genes are alternatively spliced [7] leading to substantial diversity of gene function among cells. Splicing dysregulation is implicated in cancer [8] and other diseases [9]. More broadly, mRNA-based therapeutics such as mRNA vaccines [10] are a rapidly growing area of drug development. Capturing complex aspects of mRNA biology through representation learning could accelerate scientific and therapeutic discovery, but meaningful progress requires benchmarking that reflects the unique features and functions of mRNA.

Current DNA and ncRNA benchmarks are not well-suited for mRNA biology. The regulatory language encoded in mRNAs is distinct from that of DNA and ncRNA, so performance on DNA and ncRNA-centric benchmarks may not translate to mRNA-based tasks. Salient mRNA functions are also distinct from those evaluated in ncRNA benchmarks, necessitating a novel collection of tasks that assess the representational ability of self-supervised mRNA foundational models.

To address this gap, we introduce mRNABench, a benchmarking suite designed to capture multiple facets of mRNA function and regulation. mRNABench consists of ten distinct benchmarking datasets with 59 prediction tasks, and two of these datasets – mRNA subcellular localization and eCLIP binding – are newly curated. These tasks capture the most salient aspects of mRNA biology, including transcript stability, translational efficiency, localization, and post-transcriptional regulation. On these tasks, we use linear probing to evaluate 45 self-supervised nucleotide foundation models, covering almost all publicly available models, and report insights in several key areas:

### Architectural Design:

We assess the impact of model size in nucleotide foundation models, and explore the suitability of contrastive learning and masked language modelling objectives for mRNA prediction tasks with differing contextual dependencies. Based on these findings, we add an MLM head to the Orthrus foundation model and optimally combine these objectives, achieving state-of-the-art (SOTA) performance using 700x less parameters than the best model.

### Sequence Compressibility:

We analyze the sequence content of mRNA and other genomic regions from the perspective of compression, underscoring the differences in regulatory grammar between regions. We correlate overall performance stratified by pre-training data source with these findings.

### Generalization:

As genomic benchmarking often suffers from homology-based data leakage, we use biologically-aware data-splitting strategies to assess model generalization, and report performance overestimation. Finally, we propose an approach for assessing compositional generalization in nucleotide foundation models.

Overall, our contributions include:

- mRNABench, a Python package which provides lightweight access to ten mRNA datasets with 59 function and property prediction tasks and code wrappers for 45 nucleotide foundation models. mRNABench provides integrated embedding and probing functionality, and is easily extensible to new datasets or models.
- Linear probe benchmarking of the above models on our curated datasets. We perform 135K experiments to assess the current state-of-the-art in mRNA modelling and analyze these results in terms of architecture choice, sequence compression, and generalization.
- Guided by the above experiments, we pre-train the Orthrus foundation model with an optimal mixing ratio of MLM and contrastive learning objectives to boost its original performance such that it out-competes SOTA models over 700x larger.

## 2 Related Works

### Deep learning for mRNA property prediction:

Supervised deep learning methods have long been used to predict mRNA properties from sequence. Models have been developed for key prediction tasks such as mean ribosome loading [11, 12, 13], half-life [14, 15], subcellular localization [16, 17], expression [18], and RNA-protein interaction [19, 20], highlighting the diversity of relevant tasks in mRNA biology. Most commonly, these models use a CNN-based architecture that is trained on labelled experimental data [21, 22]. While these models offer good in-distribution prediction, they are prone to overfitting on technical noise or other dataset-specific signals. Supervised learning typically has low sample-efficiency compared to transfer-learning approaches in mRNA property prediction [23], motivating self-supervised foundation models. We provide a detailed breakdown of all models evaluated in our benchmark in Appendix A.

### Benchmarks for Biological Sequences:

Several large-scale benchmarks now exist for DNA-based tasks [1, 2, 3, 4], enabling comparisons across architectures, pre-training objectives, model sizes, etc. In contrast, few RNA-focused benchmarks have been introduced [5, 6], and they primarily evaluate RNA secondary structure and function prediction in non-coding RNAs (ncRNAs). Whereas ncRNA function often depends on secondary and tertiary structures [24], mRNAs are less structured [25] and are primarily regulated through linear sequence features [26, 27, 28]. As a result, benchmarks that emphasize structural accuracy or sequence design do not capture the core biological signals relevant to mRNA biology. Existing benchmarks also use short sequences (100s of nucleotides), whereas mRNAs often span several kilobases. This introduces a gap in evaluating model performance on longer-range dependencies and full-transcript representations.

Existing benchmarks can also fail to account for data leakage due to homology between genomic sequences. This can artificially inflate generalization performance, and even common strategies such as chromosomal hold-out have shown susceptibility to homology-based leakage [29]. In mRNABench, we explicitly quantify the extent of performance overestimation due to homology and evaluate how different splitting strategies affect estimation of model generalization.

## 3 Benchmarking Tasks

We curate ten datasets, including 59 subtasks, summarized in Table 1. To our knowledge, mRNABench is the first to incorporate eight of the datasets into a comprehensive benchmark. For each dataset, we use GenomeKit [30] to generate a six-track embedding containing splice and codon positions, which has been shown to be important for predicting mRNA properties [15, 23]. We further categorize our tasks into *Global* versus *Local* based on the genomic context where the label arises from (nucleotide level vs transcript level). We briefly describe each task below, and detail the data processing pipeline in Appendix B. mRNABench can be found at: https://github.com/morrislab/mRNABench.

### 3.1 Global Tasks

**mRNA Half-Life (HL)** measures the time for half of the molecules of an mRNA transcript to degrade in the cell, and is a key determinant of transcript stability and gene expression. Longer-lived transcripts allow for more sustained protein production, whereas shorter-lived transcripts allow for rapid changes in gene expression in response to the cellular environment. We collect this data from [15], which aggregates 66 mRNA half-life experiments across human and mouse mRNA sequences.

**Mean Ribosome Load (MRL)** represents the number of ribosomes actively translating an mRNA transcript, offering a proxy for its translational efficiency. Differential translation efficiency among transcripts enables post-transcriptional regulation, as two transcripts with identical expression levels can produce vastly different amounts of protein product. Our benchmark dataset is collected from a renal cell carcinoma cell line (RCC4/VHL) [31].

**Paired mRNA Half-Life and Mean Ribosome Load (MLR-HL-Pair)** consists of synthetic mRNA sequences with paired measurements of mean ribosome load (MRL) and cellular half-life. These measurements were obtained using PERSIST-seq [32], capturing sequence variation across the 5’ UTR, CDS, and 3’ UTR, and enables joint evaluation of two key mRNA properties. With only 203 samples, this task also serves as a valuable assessment of model performance in low-data settings.

**GO Term Classification (GO):** Gene Ontology (GO) [33] provides a standardized vocabulary for describing gene and protein function across three main categories: molecular function, biological process, and cellular component. We formulate GO term prediction as a multilabel classification task, where each transcript may be associated with multiple functions. We restrict our task to a curated subset of commonly occurring terms within each ontology. Functional labels are assigned at the gene level, and the canonical isoform [34] for each gene is used as the sequence input.

**mRNA Subcellular Localization (mRNA-Loc-LR, mRNA-Loc-SR):** The subcellular compartment to which an mRNA localizes plays a crucial role in when and where its encoded protein is synthesized [35]. We processed long-read **(LR)**, isoform-resolved RNA-sequencing data from [36] , which was the first large-scale study to use direct RNA-seq for transcript localization across three compartments: Cytoplasm, Chromatin, and Polysome (active translation). We also include a short-read **(SR)** sequencing-based dataset based on APEX-seq [37], which uses an engineered enzyme to tag nearby RNAs at defined subcellular locations. This method enables mapping of RNA transcripts to eight cellular compartments. Together, these datasets offer complementary views on mRNA spatial organization.

**Protein Localization (Prot Loc)** is the subcellular localization of a protein within a cell and is a critical determinant of its function, as subcellular compartments provide distinct biochemical environments and lead to diverse interaction networks [38]. As the site of protein synthesis is directed by mRNA localization, understanding protein localization also provides insight into the spatial regulation of mRNA translation and its role in shaping cellular function. Labels for this task were drawn for the protein products of 9,769 genes determined by the Human Protein Atlas [38] across the 12 most common compartments (see Appendix B for the full list).

### 3.2 Local Tasks

**Massively Parallel Translation Assay - Mean Ribosome Load (MRL-MPRA)** is based on an MPRA from [11], in which synthetic 5’UTRs, either randomized or designed, were inserted upstream of reporter genes in human cells. The MRL was measured across multiple experimental conditions including different RNA chemistries, varying choices of UTRs, and using different reporter genes. Each subtask is framed as a regression predicting the measured mean ribosome load from sequence.

**eCLIP Binding (eCLIP):** The eCLIP protocol [39] detects the binding positions of RNA binding proteins (RBPs). RBPs regulate mRNA processing and function co- and post-transcriptionally, through alternative splicing [40], polyadenylation [41], nuclear export [42], stability [43], among others. We process an eCLIP dataset using tracks collected from ENCODE [44] covering 168 RBPs across two cell lines. From this dataset we identify the top 20 RBPs per cell line by number of events and simplify the task for linear probing by defining a binary classification task of whether each RBP binds to a given transcript.

**Variant Effect Prediction (VEP)** evaluates detection of pathogenic single-nucleotide variants (SNVs) within mature mRNA transcripts. We use a filtered subset of the TraitGym dataset [45] restricted to UTRs variants and retrieve the sequence of each sample using the APPRIS principal transcript [34]. Due the restriction of sequence input to the mature mRNA region, the inherent predictability of SNP pathogenicity may be reduced. In the future, we aim to address this through additional filtering.

## 4 Methods

### 4.1 Self-Supervised Learning Objectives

Nucleotide foundation models aim to learn useful representations of biology using self-supervised learning (SSL) on unlabelled sequence data. In this work, we focus on two common objectives: masked language modeling (MLM) and contrastive learning (CL), and later explore their combination during foundation model pre-training.

**Masked language modeling** is an SSL objective in which a model is trained to reconstruct masked-out tokens based on the surrounding sequence context. Given an input sequence x and a set of masked positions ℳ, the MLM loss is defined as: ${ℒ}_{\text{MLM}}=-\sum_{t\in ℳ}log\;p\left(x_{t}|x_{\backslash ℳ}\right)$.

**Contrastive learning learns** global representations by bringing similar sequences (e.g., augmented views) closer in embedding space while pushing dissimilar ones apart. In this work we use the Orthrus contrastive objective [23], which applies Decoupled Contrastive Learning (DCL) [46] using splice and orthogy augmentations. Given two views of the same sample $z_{i}^{1}$ and $z_{i}^{2}$ (positive pair), unrelated samples $z_{k}$, and the temperature parameter τ, the DCL objective is:

(1) $${ℒ}_{DCL,i}=log\left[\sum_{k=1}^{N}\sum_{l=1}^{2}1_{k\neq i}\;exp\left(\frac{\left\langle z_{i}^{1},\;z_{k}^{l}\right\rangle }{\tau }\right)\right]-w_{i}\frac{\left\langle z_{i}^{1},\;z_{i}^{2}\right\rangle }{\tau }$$

**Multi-task Objectives:** In Section 5.1, we explore combining MLM and CL using a simple multi-task objective. While the field of multi-objective optimization has proposed numerous strategies for multi-task optimization, recent work shows that simple scalarization (i.e. using fixed linear weights) is often the most effective in practice [47]. For the Orthrus+MLM model later described in Section 5.1, we use the pre-training objective: $ℒ=\alpha {ℒ}_{\text{MLM}}+(1-\alpha ){ℒ}_{\text{CL}}$. Here, α is a weight that balances the relative contribution of each objective, chosen based on the magnitude of the loss values at convergence in single-objective training to balance the contribution of each loss function.

### 4.2 Data Splitting Strategies

Random data splitting can overestimate model generalization due to data leakage from highly similar or homologous sequences [29]. For more rigorous assessment of generalization, mRNABench implements three biologically-informed data splitting strategies:

**Chromosomal holdout** excludes sequences from train splits based on their chromosomal origin. While this remains a common strategy in genomics, it coarsely assumes functional independence between chromosomes. Recently, Rafi et al. [29] have shown that this can cause data leakage due to sequence redundancy and homology between chromosomes.

**K-mer-based splitting** clusters sequences with similar short subsequence patterns (k-mers). We compute k-mer frequency vectors and cluster sequences using KMeans, assigning entire clusters to either split. This reduces leakage of low-level sequence motifs important for tasks like RNA-binding prediction, where much of the prediction performance is driven by the presence of these k-mers.

**Homology-based splitting** groups genes that share a common evolutionary origin. Using paralogous gene pairs retrieved from the NCBI gene table [48], we apply a 35% sequence similarity threshold to filter low-confidence relationships and build transitive gene groups such that any genes connected via paralogy are placed in the same data split. This is a stringent strategy to ensure that models are evaluated on truly unseen functional examples, though it can reduce training set diversity.

Each method has trade-offs: chromosomal holdout is the most commonly-used but most prone to data leakage, k-mer splitting reduces motif leakage but may allow some sequence-level redundancy, and homology splitting provides the most stringent generalization test but can drastically reduce training diversity. For further evaluations, we use homology splitting where possible, and naive random splitting otherwise (MRL-MPRA, MRL-HL-PAIR, VEP). We evaluate the impact of each strategy in Section 5.3.

### 4.3 Linear Probing

We use linear probing, i.e., training a linear classifier on frozen embeddings, to evaluate the representation quality of self-supervised genomic models. This strategy enables fair comparison across models ranging from millions to billions of parameters while remaining computationally tractable. Fine-tuning is sensitive to hyperparameters and model-specific, whereas linear probing offers a controlled and reproducible evaluation framework. We compute transcript-level embeddings by averaging over per-nucleotide embeddings. For models with insufficient context length, input sequences were chunked. Our experimental setup is further detailed in Appendix C.

## 5 Results

We evaluate all foundation models and two baselines on all tasks using linear probing and report the mean of results across ten random data splits in Table 2. We further train an *ab-initio* supervised CNN baseline. Prediction sub-tasks were mean-aggregated by their source dataset, and we use the best performing model from each family. In further analysis, we report a model-specific overall performance by first applying a Z-score transform to all model performances within each dataset, and then taking the mean across datasets. The Fisher transform was applied to Pearson correlations prior to Z-scoring. Full results for all models and description of baselines, chosen data split, and standard errors are reported in Appendix A and D.

**Figure 2:**
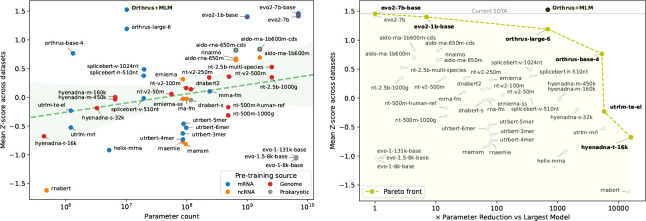
**Left:** Linear probing performance for all model variants, with the mean of z-scored metric across datasets shown on y-axis. Data point colour shows pre-training data source. **Right:** Pareto fronts showing trade-off between performance and model size. Shaded models are Pareto dominated.

We observe that Evo2 performs the best overall, while Orthrus outperforms Evo2 on global mRNA tasks and RiNALMo performs well on the MRL-MPRA task. In Figure 2, we visualize mean Z-scored metric as a function of model parameters, and observe a slight correlation between model size and overall performance, although further stratification reveals that other factors such as pre-training data source also contribute significantly to performance. We find that Orthrus and Evo2 models lie on the Pareto front of parameter efficiency, and further analyze Orthrus performance in Section 5.1.

In contrast to results from the DNA foundation modelling space [4, 45], we find that the latest generation of RNA foundation models generally do outperform simpler baseline and *ab-initio* supervised methods on most tasks. While DNA language models must contend with learning from intergenic regions with low signal-to-noise ratios, the process of alternative splicing partially alleviates this issue, and consequently makes mRNA biology a better fit for language based approaches. However, we note that the regulatory language of mRNA still differs significantly from genomic DNA, and further explore this in Section 5.2.

### 5.1 Joint pre-training objectives improve mRNA foundation models

**Figure 3:**
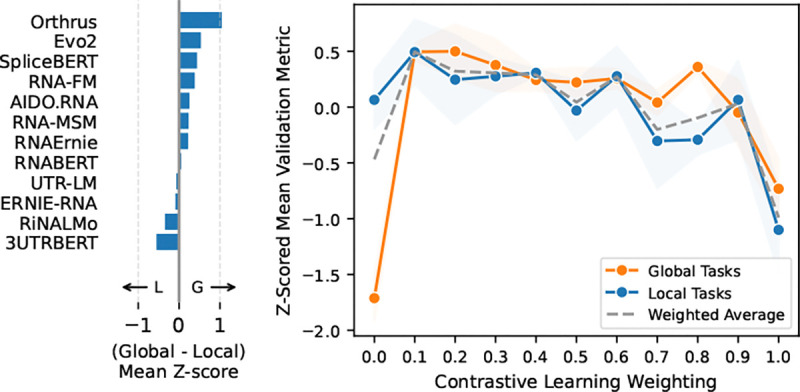
**Left:** Global task performance bias. **Right:** Performance of joint MLM-CL model as a function of contrastive objective weight.

The competitive performance of the 10M parameter Orthrus model relative to the 7B parameter Evo2 model suggests that the choice of objective function, rather than pure scale, plays a significant role in downstream performance. However, we observe that Orthrus only out-competes on global tasks, consistent with findings from the computer vision domain that contrastive pretraining objectives yield worse performance on finer-resolution tasks [49, 50]. In Figure 3 (left), we quantify each model’s global task performance bias by computing the difference in mean Z-score when grouping by task locality. We see that Orthrus over-performs on global tasks, reinforcing this known property of contrastive learning.

To address this weakness in capturing local signal, we pre-train an mRNA foundation model that combines the Orthrus contrastive objective with an MLM loss. We investigate the optimal ratio between these two objectives through scalarization [47]. To isolate the effect of training objective, we use the same pre-training dataset and Mamba backbone from Orthrus to train models with varying weightings of MLM and CL objectives. Further experimental details are described in Appendix E.

Using the joint pre-training objective in Section 4.1, we trained models with CL and MLM ratios ranging from zero (MLM-only) to one (CL-only). As seen in Figure 3 (right), CL-only models perform well on global tasks, but poorly on local tasks. Adding even a small amount of CL signal to the MLM-only model significantly improved global task performance. At low CL weights, the model’s performance on local tasks was also unchanged, indicating that CL signal can be added to boost performance on global tasks without a corresponding drop in local task performance. Overall, we find that combining MLM and CL provides better task coverage than either alone.

Using validation scores, we select the best-performing joint model, denoted **Orthrus+MLM**, and find that it beats or matches the state-of-the-art foundation model in six of ten datasets (Table 3). Orthrus+MLM also Pareto-dominates all models larger than 10M parameters (Figure 2). Surprisingly, while the addition of the MLM head boosts overall performance significantly, the gains are concentrated in global tasks, contrary to expectations, warranting further methodological exploration.

### 5.2 Sequence compressibility predicts model performance across genomic regions

**Figure 4:**
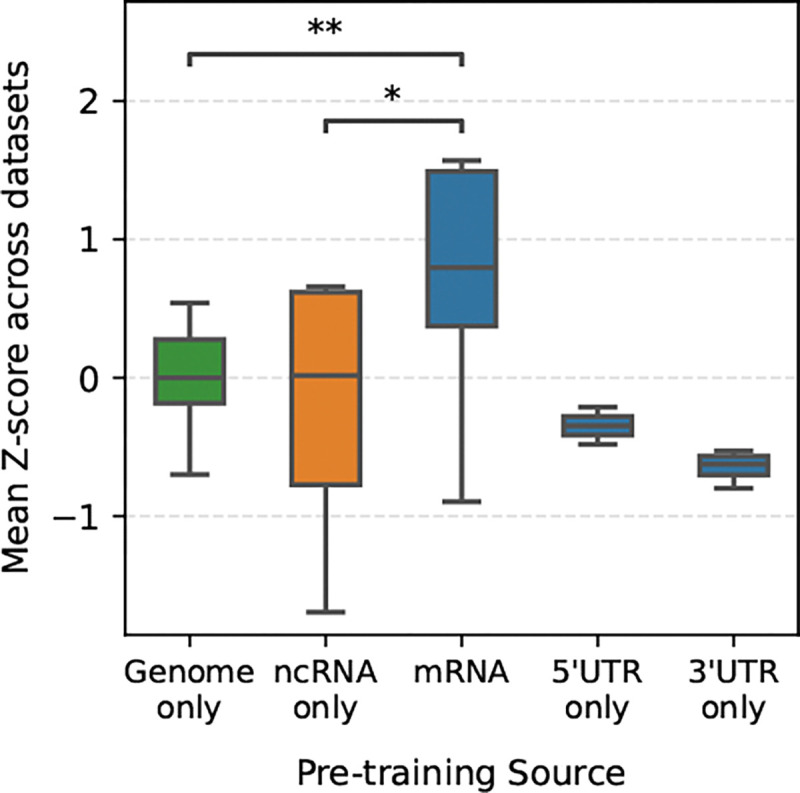
Performance stratified by pre-training data source. Significance tested using Welch’s t-test.

Although nucleotide foundation models share the same input vocabulary, the sequence content and regulatory code of the genomic regions they represent differ substantially. Consequently, we expect poor performance when applying models trained on one region (e.g., the genome) to tasks in another (e.g., mRNA), a hypothesis supported when overall model performances are stratified by pre-training dataset source (Figure 4).

In this section, we aim to quantify this distributional difference among genomic regions through their compressibility. We apply a Huffman code compressor to genomic sequences, and are able to approximate the entropy of the underlying data distribution by measuring the compressed code-length [51]. In this approach, sequences with higher frequencies of occurrence are assigned shorter code lengths, thus requiring fewer bits of information for representation. Prior work has shown that DNA and RNA are far less compressible than natural-language where higher compression ratios indicate stronger statistical regularities and underlying sequence structure [52, 53].

**Figure 5:**
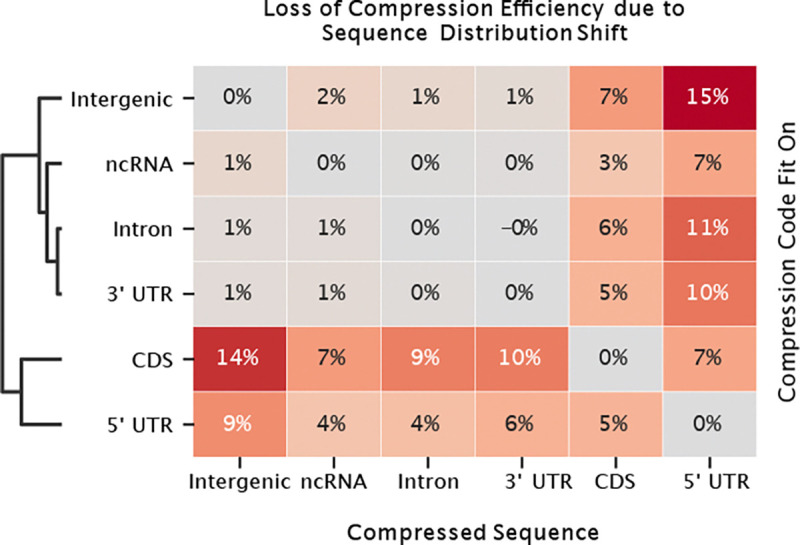
Cross-compression test scores by compression source. Numbers show percent increase in compression ratio.

We constructed distinct Huffman encoding schemes for various genomic regions: coding sequences (CDS), 5’ untranslated regions (UTRs), 3’ UTRs, non-coding RNA (ncRNA), intronic sequences, and intergenic DNA. 5’ UTR sequences produced the lowest compression ratio (0.951) followed by CDS (0.953), consistent with the additional structure imposed by codon usage and tightly regulated translation initiation sequences 8 [54, 55]. We then evaluated the efficacy of each region-specific grammar in compressing data derived from the other genomic regions. A grammar obtained from CDS compressed 3’ UTR and intergenic sequences about 10% and 14% less efficiently, indicating a significant distribution shift (Figure 5, Equation 2). In contrast, grammars originating from 3’ UTR, intergenic, intronic, and ncRNA regions compressed each other with only marginal loss in efficiency, suggesting similar sequence composition among these regions. Further analysis is described in Appendix F.

These observed compression generalization gaps highlight the heterogeneity in sequence composition across different genomic regions. This analyses provides an empirical basis for understanding the generalization challenges observed when models pre-trained on non-coding sequences are applied to mRNA specific tasks. Despite being components of mRNA, 3’ UTRs, exhibit a composition resembling intergenic sequences rather than CDS and 5’ UTR regions. This distinction likely contributes to the observed model performance discrepancies.

### 5.3 Biologically-aware data splitting offers stronger estimates of generalization

A major limitation in the evaluation of nucleotide foundation models lies in the overuse of naive random data splitting strategies, which tend to overestimate model generalization. This inflation arises because structurally or functionally related sequences often co-occur across data splits, inadvertently simplifying the predictive task. Given that functional outputs are frequently conserved among homologous or contextually similar sequences, models can achieve high performance by leveraging local sequence redundancy rather than by capturing broadly generalizable or mechanistically grounded features [29].

To evaluate the effect of data splitting strategy, we applied three biologically-informed splitting strategies across all mRNABench tasks and compared performance to random splitting. Interestingly, no single method consistently led to reduced performance across tasks. In some cases, trends aligned with biological expectations. For example, eCLIP binding performance dropped more under k-mer splitting than homology splitting, which is in line with our expectation (RBP motifs are typically between 4 – 7 nucleotides long), while GO term prediction and protein localization were more affected by homology splitting, consistent with the fact that homologous genes tend to share similar functions. Notably, these are also the tasks where our supervised baselines performed the worst, suggesting that such models are particularly sensitive to homology leakage and may rely heavily on memorized gene-level features. RNA-localization was less affected in general by all 3 splitting strategies.

### 5.4 Nucleotide foundation models perform poorly at compositional generalization

Beyond mitigating data leakage through biologically-informed splits, a deeper measure of generalization involves *systematicity*, the capacity of models to infer functional outcomes in novel combinations of familiar sequence elements. This property is central to compositional generalization, a principle widely studied in language [56] and vision [57] domains, where models are expected to generalize beyond the training distribution by recombining learned components into novel, structurally coherent configurations.

**Figure 7:**
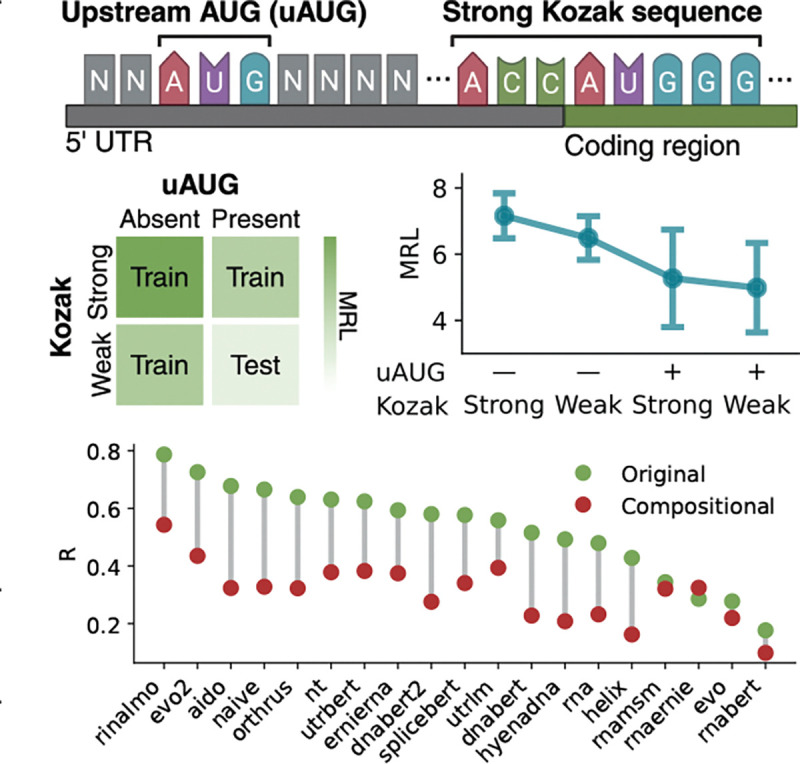
**Top:** Experimental setup. We task models with predicting MRL in sequences with upstream start codons (uAUG) and weak Kozak sequences. We expose models to either condition, but not both at once, assessing compositional generalization. **Bottom:** Performance decrease resulting from compositional split compared to original naive random split.

We extend this concept into the space of genomics, and designed a compositional generalization task to assess whether nucleotide foundation models can infer the combined effect of regulatory elements from their individual behaviours. Specifically, we focused on mean ribosome load (MRL), a proxy for translational efficiency, using a controlled subset of the MRL-MPRA dataset. Prior biological knowledge [11] indicates that upstream start codons (uAUGs) suppress MRL, while strong Kozak sequences enhance it. As shown in Figure 7 (top), we trained models on three non-overlapping subsets: (1) sequences with a strong Kozak sequence and no uAUG (positive control), (2) weak Kozak sequence and no uAUG, and (3) strong Kozak sequence with uAUG. The test set comprised sequences with a weak Kozak sequence and an uAUG, a condition never seen during training.

Our results show that models generally perform poorly at this task: linear probes on embeddings from models trained under this setup exhibit a significant drop in Pearson correlation with MRL compared to the same models trained using a naive random data split (Figure 7, bottom; full results in Appendix G). This suggests that current models do not capture the underlying structure of regulatory features interaction, pointing to a need for different inductive biases that are better aligned with the patterns present in biological sequences.

While compositional generalization is a well-established concept in language and vision, applying it to biology is more challenging. Biological motifs often co-occur and are not independent, making it harder to design clean, orthogonal feature splits. Still, the failure of current models in this controlled setting suggests that current models are not capturing the modularity of biological regulation, limiting their utility for tasks like rational sequence design where combinatorial reasoning is essential.

## 6 Conclusion

We present mRNABench, the first benchmarking suite focused on probing mature mRNA function and regulation. Spanning ten distinct datasets, 59 prediction tasks, and 135K experiments, it enables standardized evaluation across 45 nucleotide foundation models, leading to three key findings:

1. Larger models (e.g., Evo2) perform well, but Orthrus+MLM, a compact model with a biologically-informed joint objective, **matches or exceeds** their performance using over **700x fewer parameters.** This emphasizes the importance of design in addition to scaling.
2. Models trained on mRNA sequences consistently outperform those trained on DNA or ncRNA, highlighting the distributional differences between genomic regions. This is supported by compression-based analyses quantifying differences in sequence structure.
3. Naive evaluations (e.g., random splits) **overestimate generalization** and offer limited insight into what the model has learned. Using biologically-aware splits and compositional probes, we show that current models fail to capture the modular structure of mRNA regulation.

Together, these results suggest a need to move beyond solely scaling, incorporating biological priors, and designing evaluations that probe for mechanistic - rather than correlative - understanding. We do not anticipate the findings of this research to lead to negative externalities or misuse.

### Limitations and Future Work

Several limitations to our assessment exist. Due to computational limits, fine-tuning was not pursued, but may provide further insights in mRNA modelling. The assessment also omits models which performed poorly after extensive troubleshooting [58, 59], had no accessible implementations [60], or were late-breaking [61]. We envision future datasets on tasks such as 5’UTR structure [25, 62] or microRNA binding [63] prediction will also further the completeness of our tasks. Due to the extensibility of mRNABench, we envision we will be able to add these datasets in the near future.

## Figures and Tables

**Figure 6:**
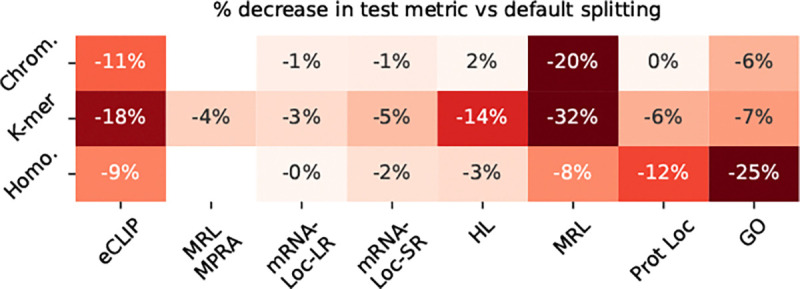
Average percent decrease in test metric across all models and sub-tasks compared to naive random splitting.

**Table 1:** Datasets included in mRNABench, grouped by task context. For benchmarking tasks with multiple subtasks, we report the range of dataset properties. For a complete breakdown see Appendix B.

Context	Benchmark	Task Type	# Unique Subtasks	# Unique Sequences	Mean Length	Max Length	Citation

Local	MRL-MPRA	Regression	6	710798	856	905	[11]
	VEP	Classification	2	4264	3659	28227	[45]
	eCLIP	Binary	40	21559	3370	205012	[44]

Global	HL	Regression	2	26662	3549	12288	[15]
	MRL	Regression	1	10,062	2620	12275	[31]
	MRL-HL-Pair	Regression	2	203	991	1585	[32]
	GO	Multilabel	3	11145	3522	12258	[33]
	Prot-Loc	Multilabel	1	9767	3592	12275	[38]
	RNA-Loc-SR	Multilabel	1	3335	4939	24319	[37]
	RNA-Loc-LR	Multilabel	1	10043	2290	13459	[36]

**Table 2:** Linear probe results. Mean of metric over ten random seeds reported. Best model per model family reported, see Appendix D for selected models. Best model for each dataset is 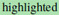 and best foundation model is **underlined**. Models not significantly worse under Wilcoxon signed-rank test at p=0.05 are **bolded**.

	Local			Global (RNA)					Global (Protein)	
	VEP	MRL MPRA	eCLIP	mRNA Loc-LR	mRNA Loc-SR	HL	MRL	MRL-HL-Pair	Prot Loc	GO
Metric	AUPRC (%)	R	AUPRC (%)	AUPRC (%)	AUPRC (%)	R	R	R	AUPRC (%)	AUPRC (%)

Naive Baseline	19.5	0.64	43.4	62.2	60.1	0.20	0.14	0.51	21.5	23.4
Naive Mamba	10.2	0.50	26.5	70.4	63.1	0.57	0.19	0.44	29.4	26.6
Supervised CNN	33.2	0.77	42.0	73.7	64.5	0.65	0.45	0.41	17.0	15.5

3UTRBERT	**31.2**	0.60	31.1	70.1	63.3	0.41	0.19	0.50	27.0	27.5
AIDO.RNA	**30.4**	0.65	41.1	77.0	73.8	0.57	0.38	0.51	38.0	42.3
DNABERT-S	**30.6**	0.52	37.4	75.3	68.4	0.47	0.29	0.39	31.1	31.8
DNABERT2	26.4	0.57	37.1	76.2	67.4	0.48	0.29	0.53	32.5	32.6
ERNIE-RNA	28.9	0.58	37.3	75.3	69.1	0.52	0.33	0.50	33.8	34.1
Evo1	12.0	0.28	26.7	70.5	63.3	0.33	0.19	0.38	29.1	27.6
Evo2	**32.1**	0.70	**47.3**	**79.5**	76.7	0.66	**0.45**	**0.51**	**40.8**	**46.7**
Helix-mRNA	7.6	0.44	29.1	73.9	61.2	0.40	0.24	0.26	29.3	25.7
HyenaDNA	23.2	0.49	37.4	76.0	67.2	0.45	0.28	**0.60**	31.8	31.0
NT	**31.8**	0.60	40.5	76.6	72.2	0.57	0.34	**0.57**	32.0	36.0
Orthrus	27.0	0.63	43.6	**79.8**	**77.9**	**0.67**	0.43	0.51	39.3	42.9
RNA-FM	25.7	0.49	35.0	74.3	67.0	0.47	0.29	0.49	32.2	32.2
RNA-MSM	16.5	0.36	28.1	72.6	63.7	0.34	0.16	0.47	29.6	27.5
RNABERT	19.2	0.24	19.2	68.3	57.5	0.21	0.06	0.32	28.0	23.8
RNAErnie	17.3	0.33	29.0	72.7	63.7	0.33	0.14	**0.55**	29.8	27.8
RiNALMo	**31.6**	**0.74**	39.2	**79.5**	69.0	0.53	0.42	0.47	32.7	34.7
SpliceBERT	28.2	0.53	37.7	76.1	72.6	0.53	0.34	**0.51**	36.0	38.2
UTR-LM	21.3	0.54	33.7	74.4	65.8	0.44	0.25	0.38	32.0	31.3

**Table 3:** Orthrus+MLM Results. Metrics reported identically to Table 2.

	VEP	MRL MPRA	eCLIP	mRNA Loc-LR	mRNA Loc-SR	HL	MRL	MRL-HL-Pair	Prot Loc	GO
Current SOTA	**32.1**	**0.74**	**47.7**	79.8	77.9	0.67	**0.45**	**0.60**	**40.8**	**46.5**
Orthrus+MLM	**32.3**	0.64	46.5	**81.2**	**78.9**	**0.70**	**0.46**	**0.63**	39.6	43.5

**Figure 8:**
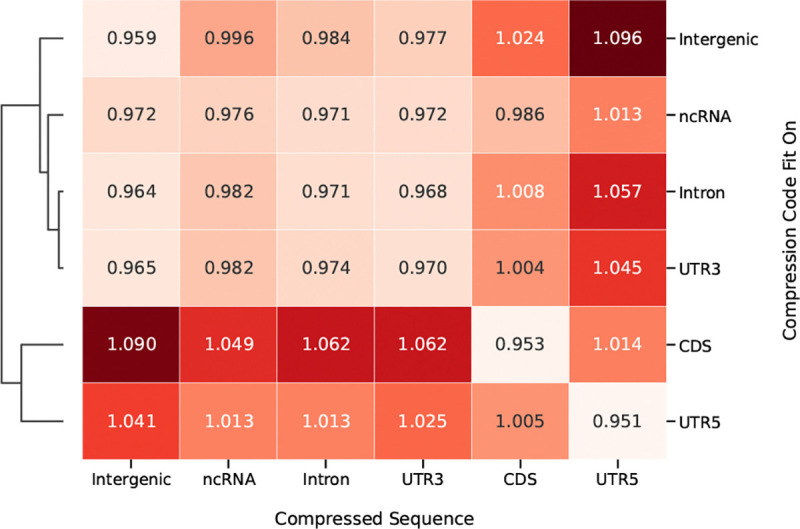
Cross-compression test scores by compression source.

**Figure 9:**
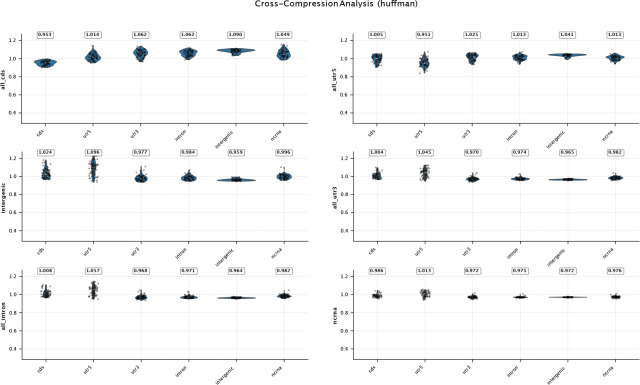
The raw distributions of 50 sequence data points compressed across Huffman codes fit to different reference sequences. 5’ UTR values demonstrate higher variability than the rest.

**Figure 10:**
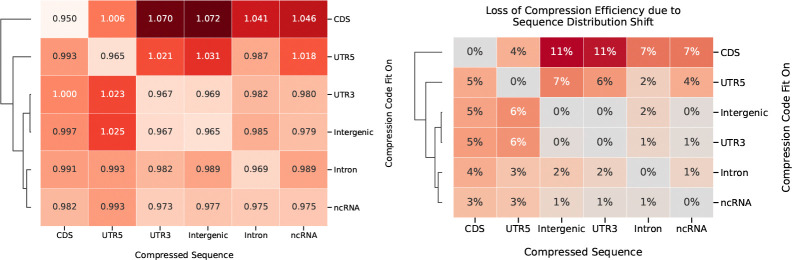
**Left:** Compression ratios indicating the percentage of size reduction associated with applying Huffman coding algorithm on the source data with k-mer length set to 6. All regions that contain repeats as indicated by repeat masker are omitted. **Right:** Cross-compression test scores by compression source. Numbers show percent increase in compression ratio while omitting genomic regions containing repeats.

## Appendix Contents

### A Overview of Evaluated Models

All evaluated nucleotide foundation models are provided below. For each model family, written in bold, several variants are usually available, which tend to vary on model size or pre-training dataset. Where available, we used the multimolecule [64] version of models.

**Table:**

Model Name	Description	Pre-train Source	# Params (M)	Pre-training Context	Citation

**3’UTRBERT**	Transformer-based mRNA model pre-trained on 3’UTR regions of 100K human mRNA sequences.	-	-	-	[65]
utrbert-3mer	3-mer tokenizer variant	mRNA	86	512	
utrbert-4mer	4-mer tokenizer variant	mRNA	87	512	
utrbert-5mer	5-mer tokenizer variant	mRNA	88	512	
utrbert-6mer	6-mer tokenizer variant	mRNA	98	512	
**AIDO.RNA**	Transformer-based RNA model trained on 42M ncRNAs fron RNACentral. Some variants domain adapted for mRNA using 9M coding sequences.	-	-	-	[66]
aido-rna-650m	-	ncRNA	650	1024	
aido-rna-1b600m	-	ncRNA	1600	1024	
aido-rna-650m-cds	CDS adaptedvariant	ncRNA, mRNA	650	1024	
aido-rna-1b600m-cds	CDS adaptedvariant	ncRNA, mRNA	1600	1024	
**DNABERT-S**	Transformer-based DNA model trained using contrastive learning on prokaryotic genomes.	Prokaryotic	117	N/A	[67]
**DNABERT2**	Transformer-based DNA model trained on multispecies dataset. Uses BPE and other modern architectural improvements for efficiency.	Genome	117	N/A	[2]
**ERNIE-RNA**	Transformer-based RNA model trained with structural information added as attention mask biases. Pretrained on 20M ncRNA sequences.	-	-	-	[68]
ernie-rna	-	ncRNA	86	1024	
ernie-rna-ss	Fine-tuned on secondary structure.	ncRNA	86	1024	
**Evol**	StripedHyena-based DNA model autoregressively trained on 300b token OpenGenome dataset.	-	-	-	[69]
evo-1–8k-base	Base model.	Prokaryotic	6453	8,192	
evo-1–131k-base	Trained with extended context.	Prokaryotic	6453	131,072	
evo-1.5–8k-base	Extends Evo1 dataset size by 50%.	Prokaryotic	6453	8,192	
**Evo2**	StripedHyena2-based DNA model autoregressively trained on the OpenGenome2 dataset, containing 9T nucleotides across all domains of life.	-	-	-	[59]
evo2–1b-base	Base model trained on mixture of genomic regions including mRNA.	Genome, ncRNA, mRNA	1000	8192	
evo2–7b-base	Base model trained on mixture of genomic regions including mRNA.	Genome, ncRNA, mRNA	7000	8,192	
evo2–7b	Extended context model trained on long genomic sequences.	Genome, ncRNA, mRNA	7000	1,000,000	
evo2–40b	Despite passing author checks, we were unable to get good performance using either 40b models.	-	-	-	
**Helix-mRNA**	mRNA foundation model trained using a Mamba2 / Transformer hybrid backbone on 26M mRNAs from eukaryotic and viral species.	mRNA	5	12,288	[70]
**HyenaDNA**	Hyena-based DNA model trained on human reference genome using an autoregressive scheme. Variants differ in size and context length.	-	-	-	[71]
hyenadna-tiny-16k	-	Genome	0.4	16,000	
hyenadna-small-32k	-	Genome	3	32,000	
hyenadna-medium-160k	-	Genome	7	160,000	
hyenadna-medium-450k	-	Genome	7	160,000	
hyenadna-large-1m	-	Genome	7	1,000,000	
**NucleotideTransformer (NT)**	Transformer-based DNA model with variants pre-trained on a differing datasets. Input tokenized to 6-mers.	-	-	-	[72]
500m-human-ref	Trained on human reference.	Genome	486	6,000	
500m-1000g	Trained on 1000 Genomes Project.	Genome	486	6,000	
2.5b-1000g	Trained on 1000 Genomes Project.	Genome	2550	6,000	
2.5b-multi-species	Trained on 850 genomes.	Genome	2550	6,000	
v2–50m-multi-species	v2 NT models use RoPE and GLUs.	Genome	56	12,288	
v2–100m-multi-species	-	Genome	98	12,288	
v2–250m-multi-species	-	Genome	235	12,288	
v2–500m-multi-species	-	Genome	498	12,288	
**Orthrus**	Mamba-based mRNA foundation model pre-trained on 40 mRNAs using contrastive learning on mRNA splice isoforms and orthologs.	-	-	-	[23]
orthrus-base-4	-	mRNA	1	12,288	
orthrus-large-6	Pre-trained using splice and codon tracks.	mRNA	10	12,288	
**RiNALMo**	Transformer-based ncRNA model trained on 36M ncRNAs. Architecture modernized to use RoPE, SwiGLU activations, and Flash Attention.	ncRNA	650	1,024	[73]
**RNABERT**	Transformer-based ncRNA model pre-trained using both MLM and a structural alignment learning objective on 80K ncRNA sequences.	ncRNA	0.5	440	[74]
**RNAErnie**	Transformer-based ncRNA model pre-trained on 23M ncRNA sequences. Uses motif-level masking during pre-training.	ncRNA	86	512	[75]
**RNA-FM**	Transformer-based RNA foundation model pre-trained on 23M ncRNA sequences. Variant mRNA-FM trained only on CDS regions.	-	-	-	[76]
rna-fm	-	ncRNA	100	1024	
mrna-fm	Trained on 45M CDS-only sequences	mRNA	239	1024	
**RNA-MSM**	Transformer-based ncRNA model trained using MSA from custom structure-based homology map on roughly 8M ncRNA sequences.	ncRNA	86	1024	[77]
**SpliceBERT**	Transformer-based mRNA model trained on 2M vertebrate mRNA sequences. Variants use only human RNA and using smaller contexts.	-	-	-	[78]
splicebert-v-1024nt	-	mRNA	20	1024	
splicebert-v-510nt	-	mRNA	19	510	
splicebert-h-510nt	Trained only on human sequences.	mRNA	19	510	
**UTR-LM**	Transformer-based mRNA model that is pre-trained on random and endogenous 5’UTR sequences from various species.	-	-	-	[79]
utrlm-te-el	Trained for translation efficiency and expression level.	mRNA	1	1026	
utrlm-mrl	Trained for mean ribosome loading.	mRNA	1	1026	

Furthermore, we also implement two naive methods. NaiveBaseline uses manually extracted sequence features as embeddings. For the naive-baseline-4 model, we take all k-mers from size three to seven as features, as well as the GC content of the sequence. The naive-baseline-6 model additionally includes the CDS length and exon count as features. The NaiveMamba model is an Mamba with three layers and 64 hidden dimensions, without any pre-training (i.e., we use the “random” initial weights). We note that this works surprisingly well as an embedding model in comparison to the earliest generation of nucleotide foundation models.

Finally, we include an *ab-initio* supervised dilated CNN comparison. This model is trained from scratch on the train split in evaluations. This supervised CNN uses a DilatedResNet architecture consiting of either three or four DilatedResNetBlocks. In each DilatedResNetBlock there are two DilatedConv1DBlocks. Each of these has two Conv1Ds layers with a kernel size of two and stride of two followed by BatchNorm1D layers, a dropout layer in-between, and residual connection followed by a MaxPool with kernel size of two. All layers are followed by ReLU activations. Each DilatedResNet block uses the same number of filters in convolutions, and has an associated dilation factor. We report these numbers for each dataset below.

For eCLIP and MRL datasets, we use four DilatedResNetBlocks with the following hyperparameters:

dilation_params: [1, 2, 4, 8]
filter_nums: [64, 128, 128, 256]

For all other tasks, we use three DilatedResNetBlocks with the following hyperparameters:

dilation_params: [1, 2, 4]
filter_nums: [64, 128, 128]

Finally, all DilatedResNets have a stochastic shift layer which randomly shuffles nucleotides by up to three positions, and the final representations are projected using two layer 256 dimension projection feed forward network.

During training, we use the AdamW optimizer with a cosine warmup of 1000 steps. The chosen learning rate was 0.001, with a weight decay of 0.00001. Gradients were clipped was used at a threshold of five. We use a batch size of 1024, trained for 5k steps on MRL tasks, 500 steps for eCLIP, and 1.5k steps for all other tasks. These experiments were run on a mixture of A100, H100, and L40 GPUs, and each run took approximately 30 minutes to run.

### B Dataset Processing Protocols

#### B.1 eCLIP

eCLIP data was downloaded from ENCODE [44]. In total, we downloaded 244 total eCLIP datasets, representing 168 unique targeted proteins; 139 of the experiments were in the K562 cell line, while the remaining 105 were in HepG2. For each eCLIP dataset, the most recent BED file was retrieved, and the corresponding BAM files from which the BED was derived were also retrieved. Given this data, we ran Peakhood [80] on both the K562 and HepG2 RBP eCLIP datasets. We retrieved a genomic annotations file (GTF) from Ensembl (release 112) [81] alongside an hg38 genomic sequences file from UCSC [82], to input as arguments to the –gtf and –gen flags, respectively. The –pre-merge flag was also used. Of the 244 total eCLIP datasets, 223 of the experiments were from a paired-end eCLIP protocol, and thus were run using only R2 reads from the eCLIP data (–bam-pp-mode 2); the remaining 21 experiments were from a single-end eCLIP protocol and were run with no BAM preprocessing (–bam-pp-mode 1). Peakhood’s output files include a set of files which identify which RNA-binding protein binding sites are associated with mature RNA transcript sequence binding (as opposed to pre-mRNA or intergenic binding sites). For this set of files, there are 2 groups, with the first group containing all possible transcript and binding site combinations and the second containing the most likely transcript sequence associated with a binding event. We chose this second group of transcript sequences as a high confidence set of sequences for the eCLIP binding task.

#### B.2 mRNA Subcellular Localization (Long Read)

The dataset used in this study [36] investigates the dynamics of RNA as it is released from chromatin, exported from the nucleus, loaded onto polysomes, and degraded in both the nucleus and cytoplasm in human cells. FASTQ files from direct RNA sequencing (library prep kit SQK-RNA002) were downloaded from GEO accession GSE208225, comprising two replicates each from four categories: cytoplasm, nucleus, poly(A), and total RNA. For analysis, we used the epi2me-labs/wf-transcriptomes pipeline via Nextflow with the flags –direct_rna True and –de_analysis. Genomic annotations (GTF) were retrieved from GENCODE release 47, and the GRCh38 primary assembly genome sequence was used as the reference genome (–ref_annotation and –ref_genome, respectively). In the sample sheet, we designated K562_cyto and K562_chr as controls, and K562_poly and K562_total as treated samples to satisfy the pipeline requirements - we did not use any differential gene analysis output. Each sample was assigned a unique barcode as required for pipeline execution. The pipeline was run with default parameters. Output included an annotated assembled transcriptome.fasta file for each sample and a transcripts_table.tsv file containing isoform-level information along with differential gene and transcript analysis results. We only retained the transcripts table for each compartment for the purposes of the benchmark.

#### B.3 mRNA Subcellular Localization (Short Read)

This dataset investigates the subcellular localization of mRNA transcripts using APEX-seq [37]. Each transcript is associated with proportions across eight cellular compartments: Nucleus, Nucleolus, Lamina, Nuclear Pore, Cytosol, ER membrane (ERM), Outer Mitochondrial Membrane (OMM), and ER Lumen. Although the original dataset was collected so that compartment labels were continuous proportions, we convert it into a multi-label classification task for simplicity in linear probing. Specifically, a compartment is considered positive if its associated proportion exceeds 0.125.

#### B.4 GO Class Prediction

Gene Ontology (GO) is a literature-curated hierarchy that assigns one or more functional terms to each gene, so predicting GO membership is inherently a multi-label classification problem. To control class imbalance while retaining biological detail, we extracted all GO terms located three levels from the root of the tree. These terms are specific enough to be informative yet common enough to provide adequate sample sizes per class. From this set, we chose the 20 most frequent terms and gathered all genes annotated with at least one of them. On average, each gene carries 1.3 of these selected labels. This design yields a task that preserves functional granularity but still offers a sufficient number of examples per class for reliable evaluation. This yields 6,722 samples for cellular components with the following GO classes:

[GO:0030426: 0, GO:0031012: 1, GO:0043025: 2, GO:0016607: 3, GO:0005635:
4, GO:0000151: 5, GO:0000786: 6, GO:0043197: 7, GO:0000781: 8,
GO:0005681: 9, GO:0032587: 10, GO:0016605: 11, GO:0036064: 12,
GO:0000776: 13, GO:0005938: 14, GO:0045202: 15, GO:0005813: 16,
GO:0009897: 17, GO:0005886: 18, GO:0090575: 19].

5,737 samples from molecular function:

[GO:0004672: 0, GO:0008201: 1, GO:0003723: 2, GO:0005102: 3, GO:0019904:
4, GO:0019899: 5, GO:0046983: 6, GO:0051015: 7, GO:0000981: 8,
GO:0005516: 9, GO:0042802: 10, GO:0020037: 11, GO:0003677: 12,
GO:0042393: 13, GO:0003714: 14, GO:0005178: 15, GO:0004888: 16,
GO:0005543: 17, GO:0044325: 18, GO:0003713: 19].

Finally 4,141 for biological process ontology hierarchies:

[GO:0007267: 0, GO:0009410: 1, GO:0001666: 2, GO:0007186: 3, GO:0016477:
4, GO:0035556: 5, GO:0007204: 6, GO:0001525: 7, GO:0098609: 8,
GO:0000226: 9, GO:0007166: 10, GO:0006897: 11, GO:0030154: 12,
GO:0007283: 13, GO:0045087: 14, GO:0030036: 15, GO:0006914: 16,
GO:0008360: 17, GO:0000278: 18, GO:0002250: 19].

We treat each of these categories as separate sub-tasks.

#### B.5 RNA Half-life

RNA half-life measures the time elapsed for half of the molecules of a given transcript to degrade. This dataset was collected from the Saluki paper [15], which itself is an aggregate of 66 experimental datasets on human and mice. We treat each species as a separate sub-task. Due to the noisiness of RNA half-life measurements, the authors of Saluki instead propose regressing on the first principal component of the experiment-by-gene matrix. We collect the RNA half-life dataset from the Orthrus [23] data release on Zenodo: https://zenodo.org/records/14708163. We converted the six-track encoding back into a nucleotide sequence, and use GenomeKit to map transcripts to its chromosome using the gene name associated with each transcript. Sequences without a gene name were dropped. See the Orthrus paper for additional details on dataset composition.

#### B.6 Mean Ribosome Load

Mean ribosome load is a measure of the average number of ribosomes which are attached to a specific transcript, which is highly correlated with the translation rate of said transcript. We collect the MRL dataset from the Orthrus [23] data release on Zenodo: https://zenodo.org/records/14708163. The original data source for this dataset can be found at [31]. We converted the six-track encoding back into a nucleotide sequence, and use GenomeKit to map transcripts to its chromosome using the gene name associated with each transcript. See the Orthrus paper for additional details on dataset composition.

#### B.7 Mean Ribosome Load - MPRA

This mean ribosome load (MRL) dataset is a massively parallel reporter assay (MPRA) that assesses the translational efficiency of random and designed 5’ UTRs. The authors insert either randomized or designed 50-nucleotide sequences into a synthetic construct and measure MRL. We collected this data from the GEO repository for [11], which provides mean ribosome load measurements associated with synthetic transcripts varying in reporter gene, RNA chemistry, and 5’ UTR sequence. Specifically, the eGFP and mCherry reporters are used; three RNA chemistries are evaluated (unmodified, pseudouridine, and m1-pseudouridine); and both random and designed 5’ UTR sequences are included. We treat each unique combination of reporter gene, chemistry, and UTR design process as a separate sub-task, averaging MRL measurements across technical replicates. Sequences lacking measurements in both replicates were excluded.

#### B.8 Paired mRNA Half-Life and MRL

We collect this data from [32], which uses PERSIST-Seq to get paired measurements of mean ribosome load and RNA half-life for a specific synthetic sequence. Also reported were various predicted measures of mRNA secondary structure, which were unused in this study but could be a future prediction target. As the source data contained sequences for the 5’UTR, CDS, and 3’UTR regions in each synthetic transcript, we were able to reconstruct the full sequence and CDS tracks. The synthetic sequences did not undergo splicing. We drop all transcripts without both a HL and MRL measurement. During evaluation, we treat the prediction of MRL and HL as separate sub-tasks.

#### B.9 Protein Localization

Protein localization reports the subcellular component that an mRNA’s protein product appears in, as reported by the Human Protein Atlas [38]. This is a multi-label classification task, as compartment labels are not mutually exclusive. The possible labels are: [Cytosol, Nucleoli, Microtubules, Vesicles, Centrosome, Mitochondria, Golgi apparatus, Nucleoplasm, Nuclear bodies, Endoplasmic reticulum, Nuclear speckles, Plasma membrane]. We collect the Prot-Loc dataset from the Orthrus [23] data release on Zenodo: https://zenodo.org/records/14708163. We converted the six-track encoding back into a nucleotide sequence, and use GenomeKit to map transcripts to its chromosome using the gene name associated with each transcript. See the Orthrus paper for additional details on dataset composition.

#### B.10 Variant Effect Prediction

Variant effect prediction (VEP) aims to classify single nucleotide polymorphisms (SNPs) as pathogenic or non-pathogenic. We use the TraitGym benchmark [45] and subset it to only SNPs that occur in 5’UTR and 3’UTR regions. The TraitGym benchmark matches positive and negative samples based on sequence context (e.g., distance to the transcription start site). TraitGym also stratifies SNPs into Mendelian and complex traits. We group sequences according to this TraitGym stratification and treat them as two separate sub-tasks. Unlike the original dataset, we provide embedding models with a SNP’s mature mRNA context rather than an unconstrained genomic context. To accomplish this, we searched the GENCODE v47 annotation using GenomeKit for mature mRNA transcripts that overlapped a specific SNP’s locus and used the principal transcript according to APPRIS to generate the sequence context for the SNP. This may limit the overall predictability of this task, as SNPs may cause pathogenic effects prior to splicing that are not detectable in mature mRNA. We aim to filter out these SNPs in the future.

### C Linear Probing Experimental Setup

We conduct linear probes by first extracting sequence embeddings using the nucleotide foundation models described in Appendix A. Generally, models will provide a per-nucleotide or per-token embedding, and we compute the mean over the sequence length to get an *H* dimensional vector per sequence. When model context length was insufficient to handle the full sequence, we computed embeddings by chunking sequences to the maximum possible length, and concatenating nucleotide embeddings across the sequence dimension prior to averaging. We found that strategies such as overlapping sequences between chunks has no major effect on performance.

Once embeddings were computed for all sequences in a dataset, we perform linear probing using Scikit-Learn’s RidgeCV for regression tasks (CV performed on train and validation splits, over values *α* = 1e-3, 1e-2, 1e-1, 1, 10), LogisticRegression for classification tasks, and a MultiOutputClassifier with LogisticRegression for multi-label tasks. The evaluation metrics are micro-averaged for multi-label tasks. For tasks (MRL) and models (NaiveBaseline) where the design matrix is too large for normal ridge regression solvers, we use the sag solver.

We replicate this linear probing across ten different data splits, using seeds [2541, 2547, 413, 412, 411, 321, 421, 2515, 2516]. To aggregate performance over datasets, we first Z-transform metrics to account for differences in metric range in various tasks. Prior to Z-transforming Pearson correlations, we apply the Fisher transform. The Z-score is computed across models for a specific seed, dataset, and sub-task. We then mean-aggregate across sub-tasks and sub-datasets (e.g., grouping targets then cell lines for eCLIP). We then report the mean of these values across seeds. In Appendix D, we also report the 95% confidence interval using the standard error. For results that are reported by model group, we select the model with best overall performance in the group for visual clarity.

Runtime for linear probes were heavily dataset and model dependent. Individual linear probes can generally be run under one hour on a standard HPC CPU node, but the embedding process is highly variable. However, for most datasets and models, the dataset embeddings were also able to be generated within an hour on either an A100 or H100. We provide functionality within our code-base to embed sequences within a specific dataset in parallel for larger datasets.

### D Full Linear Probing Results

We report the results for each model variant, for each dataset group in Table 5 and 6. For full results, we provide a parquet file in the mRNABench GitHub repository with linear probing results for all split types, seeds, models, datasets, and tasks.

**Table 5:** Full linear probing results. Metrics are mean over ten random splits, 95% confidence intervals shown.

Model	mRNA-Loc-SR Pearson R	mRNA-Loc-LR Pearson R	eCLIP AUPRC	MRL Pearson R	VEP AUPRC

aido-rna-1b600m	0.734 ± 0.008	0.772 ± 0.004	0.420 ± 0.002	0.360 ± 0.014	0.316 ± 0.032
aido-rna-1b600m-cds	0.738 ± 0.009	0.770 ± 0.005	0.411 ± 0.003	0.378 ± 0.011	0.304 ± 0.029
aido-rna-650m	0.730 ± 0.009	0.767 ± 0.005	0.408 ± 0.002	0.353 ± 0.013	0.309 ± 0.031
aido-rna-650m-cds	0.740 ± 0.007	0.771 ± 0.004	0.412 ± 0.002	0.359 ± 0.015	0.312 ± 0.036
dnabert-s	0.685 ± 0.005	0.753 ± 0.004	0.374 ± 0.002	0.292 ± 0.015	0.306 ± 0.024
dnabert2	0.675 ± 0.008	0.762 ± 0.006	0.371 ± 0.002	0.286 ± 0.021	0.264 ± 0.032
ernierna	0.691 ± 0.008	0.753 ± 0.006	0.373 ± 0.002	0.328 ± 0.017	0.289 ± 0.036
ernierna-ss	0.670 ± 0.007	0.754 ± 0.005	0.358 ± 0.003	0.293 ± 0.014	0.270 ± 0.034
evo-1–131k-base	0.633 ± 0.007	0.705 ± 0.004	0.267 ± 0.003	0.185 ± 0.017	0.120 ± 0.015
evo-1–8k-base	0.634 ± 0.008	0.706 ± 0.004	0.262 ± 0.003	0.198 ± 0.017	0.122 ± 0.018
evo-1.5–8k-base	0.633 ± 0.008	0.706 ± 0.004	0.267 ± 0.003	0.193 ± 0.013	0.123 ± 0.013
evo2–1b-base	0.770 ± 0.006	0.808 ± 0.005	0.461 ± 0.004	0.441 ± 0.013	0.319 ± 0.028
evo2–7b	0.760 ± 0.007	0.796 ± 0.004	0.477 ± 0.005	0.447 ± 0.007	0.320 ± 0.031
evo2–7b-base	0.767 ± 0.007	0.795 ± 0.004	0.473 ± 0.004	0.455 ± 0.009	0.321 ± 0.032
helix-mrna	0.612 ± 0.008	0.739 ± 0.005	0.291 ± 0.003	0.238 ± 0.018	0.076 ± 0.010
hyenadna-large-1m	0.670 ± 0.007	0.762 ± 0.005	0.374 ± 0.002	0.287 ± 0.014	0.192 ± 0.029
hyenadna-medium-160k	0.672 ± 0.009	0.760 ± 0.005	0.374 ± 0.002	0.281 ± 0.013	0.232 ± 0.026
hyenadna-medium-450k	0.674 ± 0.007	0.759 ± 0.004	0.376 ± 0.003	0.306 ± 0.012	0.240 ± 0.030
hyenadna-small-32k	0.658 ± 0.008	0.740 ± 0.005	0.353 ± 0.003	0.218 ± 0.018	0.226 ± 0.032
hyenadna-tiny-16k-d128	0.639 ± 0.006	0.724 ± 0.004	0.298 ± 0.003	0.178 ± 0.015	0.182 ± 0.031
mrna-fm	0.694 ± 0.007	0.758 ± 0.004	0.375 ± 0.002	0.321 ± 0.009	0.307 ± 0.031
naive-4	0.596 ± 0.007	0.626 ± 0.004	0.430 ± 0.004	0.143 ± 0.007	0.195 ± 0.017
naive-6	0.601 ± 0.006	0.622 ± 0.004	0.434 ± 0.004	0.145 ± 0.007	0.195 ± 0.021
naive-mamba	0.631 ± 0.006	0.704 ± 0.005	0.265 ± 0.002	0.187 ± 0.017	0.102 ± 0.014
nt-2.5b-1000g	0.699 ± 0.008	0.771 ± 0.004	0.402 ± 0.003	0.313 ± 0.024	0.298 ± 0.026
nt-2.5b-multi-species	0.722 ± 0.008	0.765 ± 0.005	0.405 ± 0.003	0.343 ± 0.012	0.318 ± 0.031
nt-500m-1000g	0.656 ± 0.008	0.739 ± 0.004	0.353 ± 0.003	0.216 ± 0.019	0.284 ± 0.020
nt-500m-human-ref	0.669 ± 0.009	0.742 ± 0.005	0.367 ± 0.004	0.249 ± 0.016	0.305 ± 0.026
nt-v2–100m-multi-species	0.704 ± 0.007	0.753 ± 0.005	0.383 ± 0.003	0.269 ± 0.016	0.292 ± 0.031
nt-v2–250m-multi-species	0.714 ± 0.009	0.763 ± 0.004	0.385 ± 0.004	0.308 ± 0.015	0.300 ± 0.035
nt-v2–500m-multi-species	0.719 ± 0.008	0.763 ± 0.005	0.391 ± 0.003	0.297 ± 0.009	0.302 ± 0.029
nt-v2–50m-multi-species	0.691 ± 0.008	0.761 ± 0.004	0.385 ± 0.004	0.269 ± 0.019	0.271 ± 0.025
orthrus-base-4	0.750 ± 0.007	0.787 ± 0.004	0.413 ± 0.003	0.363 ± 0.012	0.271 ± 0.033
orthrus-large-6	0.779 ± 0.006	0.798 ± 0.005	0.436 ± 0.004	0.427 ± 0.011	0.270 ± 0.031
orthrus-mlm	0.789 ± 0.005	0.812 ± 0.005	0.465 ± 0.003	0.460 ± 0.012	0.323 ± 0.032
rinalmo	0.690 ± 0.009	0.795 ± 0.004	0.393 ± 0.003	0.416 ± 0.021	0.316 ± 0.027
rna-fm	0.670 ± 0.006	0.743 ± 0.005	0.350 ± 0.002	0.292 ± 0.017	0.257 ± 0.036
rnabert	0.575 ± 0.006	0.683 ± 0.004	0.192 ± 0.000	0.056 ± 0.013	0.192 ± 0.025
rnaernie	0.637 ± 0.005	0.727 ± 0.004	0.290 ± 0.002	0.140 ± 0.016	0.173 ± 0.027
rnamsm	0.637 ± 0.006	0.726 ± 0.005	0.281 ± 0.002	0.157 ± 0.016	0.165 ± 0.035
splicebert-h-510nt	0.720 ± 0.009	0.750 ± 0.003	0.359 ± 0.002	0.297 ± 0.015	0.270 ± 0.034
splicebert-v-1024nt	0.726 ± 0.010	0.761 ± 0.004	0.377 ± 0.002	0.336 ± 0.013	0.282 ± 0.033
splicebert-v-510nt	0.679 ± 0.007	0.733 ± 0.003	0.331 ± 0.002	0.238 ± 0.014	0.263 ± 0.032
supervised-cnn	0.645 ± 0.005	0.737 ± 0.009	0.421 ± 0.005	0.448 ± 0.021	0.332 ± 0.023
utrbert-3mer	0.629 ± 0.008	0.709 ± 0.006	0.296 ± 0.002	0.157 ± 0.014	0.300 ± 0.035
utrbert-4mer	0.623 ± 0.013	0.699 ± 0.005	0.286 ± 0.001	0.156 ± 0.016	0.297 ± 0.028
utrbert-5mer	0.639 ± 0.009	0.720 ± 0.004	0.313 ± 0.002	0.171 ± 0.017	0.297 ± 0.034
utrbert-6mer	0.633 ± 0.011	0.701 ± 0.005	0.311 ± 0.002	0.191 ± 0.015	0.312 ± 0.029
utrlm-mrl	0.648 ± 0.007	0.732 ± 0.004	0.308 ± 0.001	0.223 ± 0.015	0.131 ± 0.024
utrlm-te-el	0.658 ± 0.007	0.743 ± 0.005	0.337 ± 0.002	0.249 ± 0.012	0.213 ± 0.025

**Table 6:** Full linear probing results. Metrics are mean over ten random splits, 95% confidence intervals shown.

Model	HL Pearson R	MRL-HL-Pair Pearson R	MRL-MPRA Pearson R	GO AUPRC	Prot-Loc AUPRC

aido-rna-1b600m	0.555 ± 0.010	0.534 ± 0.074	0.633 ± 0.001	0.366 ± 0.004	0.343 ± 0.005
aido-rna-1b600m-cds	0.567 ± 0.009	0.511 ± 0.044	0.645 ± 0.001	0.423 ± 0.004	0.380 ± 0.006
aido-rna-650m	0.542 ± 0.010	0.503 ± 0.060	0.645 ± 0.001	0.366 ± 0.004	0.349 ± 0.003
aido-rna-650m-cds	0.561 ± 0.010	0.489 ± 0.044	0.654 ± 0.001	0.419 ± 0.003	0.377 ± 0.004
dnabert-s	0.467 ± 0.007	0.393 ± 0.035	0.516 ± 0.001	0.318 ± 0.004	0.311 ± 0.004
dnabert2	0.475 ± 0.007	0.534 ± 0.050	0.570 ± 0.001	0.326 ± 0.003	0.325 ± 0.005
ernierna	0.518 ± 0.009	0.503 ± 0.074	0.580 ± 0.002	0.341 ± 0.003	0.338 ± 0.004
ernierna-ss	0.456 ± 0.011	0.532 ± 0.055	0.506 ± 0.002	0.324 ± 0.004	0.322 ± 0.005
evo-1–131k-base	0.331 ± 0.009	0.383 ± 0.061	0.276 ± 0.002	0.277 ± 0.004	0.291 ± 0.003
evo-1–8k-base	0.329 ± 0.007	0.330 ± 0.049	0.274 ± 0.002	0.278 ± 0.004	0.295 ± 0.004
evo-1.5–8k-base	0.322 ± 0.009	0.352 ± 0.073	0.271 ± 0.002	0.279 ± 0.004	0.295 ± 0.003
evo2–1b-base	0.639 ± 0.010	0.572 ± 0.053	0.666 ± 0.001	0.458 ± 0.003	0.399 ± 0.008
evo2–7b	0.653 ± 0.009	0.527 ± 0.077	0.699 ± 0.001	0.465 ± 0.003	0.404 ± 0.009
evo2–7b-base	0.661 ± 0.007	0.507 ± 0.066	0.705 ± 0.001	0.467 ± 0.003	0.408 ± 0.007
helix-mrna	0.400 ± 0.010	0.261 ± 0.070	0.439 ± 0.002	0.257 ± 0.003	0.293 ± 0.005
hyenadna-large-1m	0.462 ± 0.007	0.506 ± 0.060	0.501 ± 0.001	0.308 ± 0.004	0.311 ± 0.005
hyenadna-medium-160k	0.446 ± 0.009	0.603 ± 0.060	0.493 ± 0.001	0.310 ± 0.004	0.318 ± 0.005
hyenadna-medium-450k	0.459 ± 0.009	0.460 ± 0.059	0.480 ± 0.001	0.316 ± 0.004	0.316 ± 0.004
hyenadna-small-32k	0.428 ± 0.008	0.625 ± 0.037	0.469 ± 0.002	0.306 ± 0.004	0.315 ± 0.005
hyenadna-tiny-16k-d128	0.390 ± 0.008	0.440 ± 0.071	0.424 ± 0.002	0.283 ± 0.004	0.303 ± 0.004
mrna-fm	0.546 ± 0.011	0.252 ± 0.068	0.011 ± 0.003	0.404 ± 0.003	0.356 ± 0.004
naive-4	0.143 ± 0.012	0.510 ± 0.078	0.642 ± 0.001	0.231 ± 0.004	0.214 ± 0.005
naive-6	0.198 ± 0.011	0.511 ± 0.073	0.642 ± 0.001	0.234 ± 0.004	0.215 ± 0.005
naive-mamba	0.574 ± 0.005	0.437 ± 0.047	0.499 ± 0.001	0.266 ± 0.004	0.294 ± 0.004
nt-2.5b-1000g	0.499 ± 0.008	0.560 ± 0.051	0.554 ± 0.001	0.341 ± 0.003	0.331 ± 0.006
nt-2.5b-multi-species	0.570 ± 0.008	0.567 ± 0.053	0.602 ± 0.002	0.360 ± 0.003	0.320 ± 0.006
nt-500m-1000g	0.402 ± 0.007	0.511 ± 0.063	0.446 ± 0.002	0.304 ± 0.004	0.310 ± 0.005
nt-500m-human-ref	0.454 ± 0.008	0.569 ± 0.055	0.463 ± 0.002	0.303 ± 0.004	0.288 ± 0.006
nt-v2–100m-multi-species	0.488 ± 0.007	0.516 ± 0.054	0.507 ± 0.001	0.337 ± 0.004	0.330 ± 0.006
nt-v2–250m-multi-species	0.514 ± 0.008	0.535 ± 0.047	0.577 ± 0.001	0.339 ± 0.005	0.335 ± 0.008
nt-v2–500m-multi-species	0.530 ± 0.007	0.512 ± 0.051	0.571 ± 0.001	0.350 ± 0.003	0.336 ± 0.006
nt-v2–50m-multi-species	0.480 ± 0.008	0.487 ± 0.047	0.510 ± 0.002	0.326 ± 0.003	0.324 ± 0.006
orthrus-base-4	0.542 ± 0.009	0.544 ± 0.061	0.617 ± 0.001	0.378 ± 0.005	0.364 ± 0.006
orthrus-large-6	0.667 ± 0.005	0.513 ± 0.089	0.629 ± 0.001	0.429 ± 0.004	0.393 ± 0.006
orthrus-mlm	0.696 ± 0.005	0.633 ± 0.047	0.639 ± 0.001	0.435 ± 0.005	0.396 ± 0.005
rinalmo	0.532 ± 0.009	0.466 ± 0.066	0.742 ± 0.001	0.347 ± 0.005	0.327 ± 0.004
rna-fm	0.472 ± 0.007	0.494 ± 0.071	0.487 ± 0.001	0.322 ± 0.004	0.322 ± 0.005
rnabert	0.206 ± 0.009	0.322 ± 0.065	0.236 ± 0.002	0.238 ± 0.004	0.280 ± 0.005
rnaernie	0.334 ± 0.008	0.550 ± 0.046	0.325 ± 0.002	0.278 ± 0.004	0.298 ± 0.005
rnamsm	0.340 ± 0.010	0.474 ± 0.042	0.357 ± 0.002	0.275 ± 0.004	0.296 ± 0.005
splicebert-h-510nt	0.524 ± 0.009	0.512 ± 0.058	0.567 ± 0.001	0.379 ± 0.004	0.356 ± 0.005
splicebert-v-1024nt	0.531 ± 0.007	0.514 ± 0.056	0.533 ± 0.002	0.382 ± 0.003	0.360 ± 0.005
splicebert-v-510nt	0.462 ± 0.008	0.575 ± 0.059	0.518 ± 0.001	0.327 ± 0.004	0.326 ± 0.005
supervised-cnn	0.647 ± 0.004	0.411 ± 0.083	0.771 ± 0.008	0.155 ± 0.002	0.169 ± 0.006
utrbert-3mer	0.407 ± 0.011	0.502 ± 0.056	0.556 ± 0.001	0.265 ± 0.004	0.265 ± 0.005
utrbert-4mer	0.395 ± 0.011	0.430 ± 0.060	0.560 ± 0.002	0.267 ± 0.002	0.270 ± 0.004
utrbert-5mer	0.405 ± 0.013	0.471 ± 0.053	0.572 ± 0.001	0.296 ± 0.004	0.298 ± 0.003
utrbert-6mer	0.407 ± 0.010	0.497 ± 0.055	0.595 ± 0.002	0.275 ± 0.004	0.270 ± 0.003
utrlm-mrl	0.387 ± 0.008	0.412 ± 0.078	0.538 ± 0.001	0.293 ± 0.004	0.310 ± 0.004
utrlm-te-el	0.444 ± 0.009	0.379 ± 0.070	0.540 ± 0.002	0.313 ± 0.004	0.320 ± 0.005

### E Joint Objective Experimental Setup

We perform an ablation study on the mixture of contrastive learning (CL) and masked language modelling (MLM) losses using the Orthrus model as a controlled architecture. Here, we use the architecture from the orthrus-large-6 model, which is a Mamba model with six layers and a hidden dimension of 512. See the Orthrus paper for further optimizer hyperparameters, which we matched exactly. We use a subset of the Orthrus pre-training dataset, which is uses RefSeq annotations for ten species to generate positive pairs for contrastive learning. In total, this results in approximately 1M mRNA sequences.

To ablate the balance of the MLM and CL objectives, we first trained models with only MLM and CL, and assessed the gradient magnitude upon convergence. We then selected values of *α* for the equation in Section 5.1 balancing MLM and CL such that the converged gradient magnitude for MLM and CL would be equal when *α* = 0.5. We then interpolated *α* from 0 to 1 in increments of 0.1. For each value of *α*, we pre-train three models using different seeds, for a total of 30 models. Each Orthrus pre-training run took approximately 16 hours using 4 H100 GPUS. We then select the best model for further evaluation using a simple weighted average of validation loss between global and local tasks, which was the *α* = 0.1 model. Here, the validation loss was computed similarly to other overall metric aggregations, including the use of identical random data splitting seeds. Finally, we note that the shaded area shown in Figure 3 (right) shows standard error of these mean validation losses.

### F Genomic Data Compression

We define the Compression Ratio CR for a given sequence s using a specific compression model M. This model M (e.g., a Huffman codebook) is typically derived from training on a representative set of sequences from a particular source distribution (e.g., CDS, 5’ UTRs). The CR quantifies the efficiency of the compression by comparing the total number of bits required to represent the sequence using model M to its original size.

Let the sequence s consist of N=|s| tokens (e.g., k-mers), denoted as $s^{1},s^{2},\ldots ,s^{N}$, $L_{M}=\left(s^{k}\right)$ be the code length (in bits) assigned to the kth token $s^{k}$ by the model M. Let $b_{\mathrm{original}}$ be the number of bits used to represent each token in the original, uncompressed fixed-length format (e.g., 2×kmer length for DNA k-mers). The Compression Ratio is then:

(2)
$$CR(s,M)=\frac{\sum_{k=1}^{N}L_{M}\left(s^{k}\right)}{N\cdot b_{\mathrm{original}}}$$

The numerator represents the total compressed size of the sequence s in bits when encoded using model M. The denominator represents the total original size of the sequence in bits. A lower CR indicates better compression, reflecting higher statistical regularity in the sequence s as captured by the model M. This normalized metric facilitates comparisons of compressibility across different sequences and data domains.

When comparing two different compression models, $M_{l}$ and $M_{m}$, fit on distinct data sources (e.g., source l and source m), we can assess their relative effectiveness on a specific sequence s. If s originates from the same source distribution as the training data for $M_{m}$, the difference:

(3)
$$\Delta CR=CR\left(s,M_{l}\right)-CR\left(s,M_{m}\right)$$

quantifies the loss in compression efficacy when using a model trained on a different data source $M_{l}$ compared to the model trained on the native source $M_{m}$. A positive ΔCR indicates that model $M_{l}$ is less efficient for sequence s, suggesting a quantifiable difference between the underlying statistical distributions of the data sources l and m.

**Algorithm 1:** Huffman Coding Pseudocode

**Require:** Symbols $X=\left\{x_{1},\ldots ,x_{n}\right\}$ with frequencies $F=\left\{f_{1},\ldots ,f_{n}\right\}$	
1:	**function** Huffman(X,F)
2:	Q ← min-heap containing one leaf $\left(x_{k},f_{k}\right)$ for each k
3:	**while** |Q|>1 **do**
4:	$\left(a,\;f_{a})\;\leftarrow \;Q.extractMin(\right)$
5:	$\left(b,\;f_{b})\;\leftarrow \;Q.extractMin(\right)$
6:	$Q.insert(Node(a,\;b,\;f_{a}\;+\;f_{b}))$
7:	**end while**
8:	root←Q.extractMin()
9:	codes ← dict{}
10:	ASSIGN(root, "")
11:	**return** codes
12:	**end function**
13:	
14:	**function** ASSIGN(node, prefix)
15:	**if** isLeaf(node) **then**
16:	codes[node.symbol] ← prefix
17:	**else**
18:	ASSIGN(node.left, prefix + 0)
19:	ASSIGN(node.right, prefix + 1)
20:	**end if**
21:	**end function**

For the compression algorithm we utilize Huffman coding, shown in Algorithm 1, which is a widely used algorithm for lossless data compression. Its strategy is to assign shorter binary codes to more frequent symbols and longer codes to less frequent ones, thereby minimizing the average code length for a given text or data sample.

Specifically, Huffman compression relies on empirically estimating the probability of discrete events from a given data sample. Based on these estimated probabilities, the algorithm assigns variable length binary codes to each event. The length of these codes is inversely proportional to the symbol’s probability: more frequent symbols receive shorter codes. For an event $x_{i}$ with probability $p\left(x_{i}\right)$, the optimal code length is $-{log}_{2}\;p\left(x_{i}\right)$ bits. Huffman coding aims to construct a prefix code that approaches this theoretical optimum in terms of average code length.

Let $P_{M}$ be the probability distribution of tokens (symbols) implicitly learned by a compression model M during its training (e.g., from the frequencies of k-mers in a training sequence). The average code length assigned by model M to tokens from this distribution $P_{M}$, denoted $L_{M}$, satisfies $H\left(P_{M}\right)\leq L_{M}<H\left(P_{M}\right)+1$, where $H\left(P_{M}\right)$ is the Shannon entropy of the source distribution $P_{M}$:

(4)
$$H\left(P_{M}\right)=-\sum_{x\in X}P_{M}(x){\;log}_{2}\;P_{M}(x)$$

Here, X is the set of all possible tokens. This indicates that Huffman coding is nearly optimal for the data distribution it was trained on.

Similarly how compression ration CR of a sequence s has a close connection to the underlying entropy of a distribution, we can draw a similar connection to measuring cross entropy between two distributions. This is done by applying a compression model M fit on $P_{m}$ to compress a sequence sampled $s_{T}$ from an underlying probability distribution $P_{T}$.

Consider two probability distributions defined over the same sample space $P_{M}$ and $P_{T}$. Assume sequence $s_{T}$ is drawn from $P_{T}$. When we use a compression model M (which was fit on $P_{M}$) to compress $s_{T}$, the average number of bits per token used to encode $s_{T}$ is

(5)
$$\frac{1}{N}\sum_{k=1}^{N}L_{M}\left(s_{T}^{k}\right).$$

This empirical average code length is an estimate of the cross-entropy between the true distribution $P_{T}$ and the model’s distribution $P_{M}$:

(6)
$$H\left(P_{T},P_{M}\right)=-\sum_{x\in X}P_{T}\left(x\right)\;{log}_{2}\;P_{M}(x)$$

The term $L_{M}\left(s^{k}\right)$ represents the code length assigned by model M to the specific token $s^{k}$. In the case of using Huffman coding compression $P_{M}(x)$ are the probabilities used to construct the Huffman codes in model M, then $L_{M}(x)\approx -{log}_{2}\;P_{M}(x)$ for each token x.

The numerator of the Compression Ratio CR 2 is the total compressed size of sequence s using model M. Thus, $CR\left(s_{T},M\right)\cdot b_{\mathrm{original}}=\frac{1}{N}\sum_{k=1}^{N}L_{M}\left(s^{k}\right)$, which is the empirical average bits per token. Therefore, the Compression Ratio $CR\left(s_{T},M\right)$ is directly proportional to this empirical estimate of $H\left(P_{T},P_{M}\right)$.

It is important to note that this analysis, based on token frequencies and Huffman coding, has inherent limitations. The choice of token size (e.g., k-mer length) is critical: larger tokens can capture more local context but may result in an exponentially larger sample space. This can make it challenging to empirically estimate the true probability distributions from finite training data, potentially leading to inaccurate frequency counts for rare events. In addition certain structures within the genome have a specific repetition order such as an open reading frame in coding sequences consisting of a sequence of codons composed of three nucleotides each. We use k-mer length of six to capture this regularity.

Furthermore, by focusing on individual token frequencies, this approach largely ignores global sequence structure and long-range dependencies. Such larger-scale structures, including regulatory motifs, codon usage patterns beyond local k-mers, and repetitive elements, are known to be significant features in both coding and intergenic regions and are not directly captured by this type of local, frequency-based compression analysis.

Training data for the compression analysis was generated using Genome Karyotype (genome-kit) [30] and GENCODE Basic version 47 annotations [83]. From these annotations, we isolated 3’ UTRs, 5’ UTRs, introns, and coding sequences (CDS) of protein-coding genes.

Because intergenic regions are vast, we restricted our intergenic sequence set to those on chromosome 1, which is about 10% of the human genome. This selection helped balance the corresponding length of different genomic region types in our training data, reducing potential biases in the compression model fitting that could arise (Table 7). For genes with multiple transcripts, we selected the canonical isoform using the APPRIS database [34] to ensure consistency and reduce redundancy from alternative splice variants.

**Table 7:** Distribution and characteristics of sequences with and without repeats across various genomic regions. Counts represent the number of distinct sequences. Lengths are reported in base pairs (bp).

Genomic Region	Num. Sequences with Repeats	Total Length with Repeats (bp)	Num. Sequences without Repeats	Total Length without Repeats (bp)

CDS	3,706	4,927,801	3,121	3,883,277
Intron	2,922	91,533,879	415	569,014
UTR3	3,506	5,271,453	2,241	1,902,431
UTR5	3,499	629,402	3,127	496,448
Intergenic(chr1)	196	13,640,727	4,157	148,795,829*
ncRNA	38,016	33,615,313	14,648	9,549,965

The number of bases used for estimating the compression is omitting repeats and (*) indicates that we use fewer bases for computational efficiency.

When preparing training data for each category, we excluded a hold-out set of 50 unique sequences of variable lengths. These sequences were never seen by the compression algorithm during its fitting stage and were used as a test set. For the Huffman compression algorithm, all sequences (training and test) were first divided into non-overlapping k-mers of length six. Sequences were then truncated at their 3’ end to ensure their total length was a multiple of six, losing at most five nucleotides per sequence. This preprocessing was crucial to keep the reading frame intact when sequences within each category were combined for frequency analysis by the compression algorithm.

Finally, to check if repetitive elements affected our findings, we conducted a parallel analysis where repeat regions, identified by RepeatMasker [84], were excluded from all genomic categories. The results from this repeat-masked analysis were very similar to those in the main paper, suggesting common repeats did not significantly affect our conclusions (Figure 10).

### G Compositional Generalization Setup and Results

To construct a task for compositional generalization, we use the MRL-MPRA dataset [11] which measures MRL from synthetically generated 5’ UTRs. We scan each 5’ UTR for the presence of an upstream start codon (uAUG) and the strength of its Kozak sequence. The Kozak sequence, also known as the translation intiation start site, is a primary factor in ribosome loading and transitation initiation. We use the reported nucleotide frequencies of the 20 most and 20 least repressive sequences from this dataset to classify each 5’ UTR as having a strong or weak Kozak sequence. A sequence is classified as strong or weak only if the nucleotide at each of positions −1, −2, and −3 (relative to the start codon) belongs to the specific set of allowed nucleotides for that position as listed in Table 8. If any position contains a nucleotide outside its allowed set, the sequence is classified as mixed.

**Table 8:** Classification of Kozak sequences as strong or weak. −1 is the nucleotide position relative to the start codon.

Kozak sequence strength	−3	−2	−1

Strong	A, G	C, A	C, G, A
Weak	T	G	T, C

We split the data into four subsets: (1) strong Kozak without an uAUG, (2) weak Kozak without an uUAG, (3) strong Kozak with an uAUG, and (4) weak kozak with an uAUG. We train on (1)-(3) and test on the unseen combination (4).

The model-specific gaps between the Pearson R when using the default split versus the compositional split are reported in Table 9.

**Table 9:** Compositional generalization gap for all models. Reported values are the difference between Pearson R for the default split and compositional split.

Model	Drop in R

aido-rna-1b600m	−0.30
aido-rna-1b600m-cds	−0.35
aido-rna-650m	−0.30
aido-rna-650m-cds	−0.36
dnabert-s	−0.29
dnabert2	−0.30
ernierna	−0.22
ernierna-ss	−0.26
evo-1-131k-base	−0.08
evo-1-8k-base	−0.06
evo-1.5-8k-base	−0.13
evo2-1b-base	−0.27
evo2-7b	−0.29
helix-mrna	−0.27
hyenadna-large-1m	−0.29
hyenadna-medium-160k	−0.28
hyenadna-medium-450k	−0.28
hyenadna-small-32k	−0.25
hyenadna-tiny-16k-d128	−0.24
naive-4	−0.34
naive-6	−0.34
nt-2.5b-1000g	−0.23
nt-2.5b-multi-species	−0.25
nt-500m-1000g	−0.17
nt-500m-human-ref	−0.20
nt-v2-100m-multi-species	−0.17
nt-v2-250m-multi-species	−0.22
nt-v2-500m-multi-species	−0.23
nt-v2-50m-multi-species	−0.23
orthrus-base-4	−0.33
orthrus-large-6	−0.32
rinalmo	−0.24
rna-fm	−0.25
rnabert	−0.08
rnaernie	0.04
rnamsm	−0.02
splicebert-h-510nt	−0.24
splicebert-v-1024nt	−0.24
splicebert-v-510nt	−0.22
utrbert-3mer	−0.24
utrbert-4mer	−0.29
utrbert-5mer	−0.27
utrbert-6mer	−0.24
utrlm-mrl	−0.33
utrlm-te-el	−0.17
